# Echocardiographic follow-up after cardiac rehabilitation designed for patients with obesity

**DOI:** 10.1007/s10554-023-02805-1

**Published:** 2023-03-16

**Authors:** Sanne M. Snelder, Iris den Uijl, Madoka Sunamura, Felix Zijlstra, Nienke ter Hoeve, Bas M. van Dalen

**Affiliations:** 1 Department of Cardiology, Franciscus Gasthuis & Vlietland, Rotterdam, the Netherlands; 2 Capri Cardiac Rehabilitation, Rotterdam, the Netherlands; 3 Department of Rehabilitation Medicine, Erasmus University Medical Center Rotterdam, Erasmus MC, Rotterdam, the Netherlands; 4 Department of Cardiology, Thoraxcenter, Erasmus University Medical Center Rotterdam, Erasmus MC, Rotterdam, the Netherlands

**Keywords:** Obesity/Obese, Cardiac rehabilitation, Echocardiography, Global longitudinal strain

## Abstract

We hypothesize that a novel tailor-made cardiac rehabilitation (CR) program for obesity patients (OPTICARE XL) has better outcomes as compared to usual CR regarding parameters of cardiac function as measured by conventional and advanced transthoracic echocardiography. This is an open-label, randomized controlled trial. Inclusion criteria were: patients referred to CR with a body mass index (BMI) ≥30 kg/m2, and age ≥18 years with either coronary artery disease or nonvalvular atrial fibrillation. The experimental group participated in OPTICARE XL and the controls received the usual CR. Subjects randomized to OPTICARE XL received on top of usual CR behavioural therapy for a healthy diet and an active lifestyle for the first 12 weeks. Also, the exercise program was more tailored. Furthermore, a behavioural after-care program was organized with 6 meetings between weeks 13-52. Transthoracic (speckle tracking) echocardiography was performed at baseline and one-year follow-up. A total of 42 patients completed the follow-up, 21 in both groups. There was a mild but statistically significant reduction in weight over time, however, this was comparable between groups. There was no improvement observed in any of the echocardiographic parameters. In conclusion, cardiac function in obesity patients was not improved one-year after a novel tailor-made CR program (OPTICARE XL) as compared to usual CR.

## Condensed Abstract

We hypothesized that a novel state of the art cardiac rehabilitation program designed for patients with obesity has better outcomes as compared to standard CR regarding parameters of cardiac function as measured by transthoracic echocardiography. However, cardiac function in patients with obesity did not improve as compared to standard CR.

## Introduction

Cardiac rehabilitation (CR) is a valuable treatment for patients with a broad spectrum of cardiac disease. Currently, CR has a class 1 recommendation (evidence and/or general agreement that a given treatment or procedure is beneficial, useful, effective) in several European society of cardiology (ESC) and American college of cardiology (ACC) guidelines [1–3]. Throughout the years, CR has evolved from exercise only into a comprehensive program that also addresses other cardiovascular disease risk factors and provides education, includes social support and focusses on lifestyle [4–6]. By implementing these progresses, sustained improvements were made not only in social and physical functioning but also in hospitalization and mortality rates at 5-years [7–10].

Currently, at entry into CR over 80% of the patients are overweight (body mass index (BMI) ≥ 25 kg/m^2^), and 38% are even obese (BMI ≥ 30 kg/m^2^) [11]. However, standard CR programs are far from optimal in patients with obesity, who have a higher cardio-metabolic risk and poorer fitness [5, 11]. There is growing evidence that effects achieved during standard CR in patients with obesity are substantially smaller than in non-obese patients [12]. Nevertheless, intentional weight loss, accomplished through behavioural weight loss and exercise, improves insulin sensitivity and associated cardio-metabolic risk factors such as lipid measures, blood pressure, inflammation, and vascular function [13]. However, standard CR programs are lacking behavioural weight loss programs tailored for patients with obesity [14, 15].

Recently, we have shown that weight loss achieved by bariatric surgery improves many echocardiographic parameters of cardiac function and dimension at one-year follow-up [16, 17]. Besides the benefits of weight reduction, exercise itself could reverse parameters of cardiac function measured with (advanced) echocardiography 18] Therefore, we hypothesized that a novel state of the art CR program for patients with obesity (OPTICARE XL CR) with a focus on exercise and behavioural weight loss has better outcomes as compared to standard CR regarding conventional and advanced transthoracic echocardiography parameters of cardiac function.

## Methods

### Study population and design

The OPTICARE XL CR (OPTImal CArdiac REhabilitation XL CR) study is an open-label, randomized controlled trial [19] Inclusion criteria were: patients with a BMI ≥ 30 kg/m^2^ and age ≥ 18 years with either coronary artery disease (myocardial infarction, angina pectoris) or nonvalvular atrial fibrillation, who were referred to CR. Exclusion criteria were: heart failure, left ventricle (LV) ejection fraction < 40%, implantable cardioverter defibrillator, psychological or cognitive impairments, renal failure or other severe co-morbidity (e.g. severe chronic obstructive pulmonary disease, active malignancy, poorly controlled diabetes, intermittent claudication, and musculoskeletal impairments) which could impair participation in CR. The current study concerns a substudy of the OPTICARE XL trial. After inclusion in OPTICARE XL CR, consecutive patients were asked to participate in this echocardiography substudy. Inclusion occurred only during a pre-determined period and at only one inclusion site.

The experimental group participated in OPTICARE XL CR, and the controls received standard CR as recommended by the guidelines [20] Standard CR lasted 6–12 weeks and comprised two group exercise sessions per week complemented with general lifestyle and cardiovascular risk factor education and counselling. Subjects randomized to OPTICARE XL CR received behavioural therapy for a healthy diet (once a week program of 60 min during 12 weeks) and an active lifestyle (once every 3 weeks a program of 45 min during 12 weeks). The exercise program during the first 12 weeks was a combination of aerobic training and muscle strength training. This combination is considered to be the preferred training in patients with obesity [21].

In order to enter cardiac rehabilitation, patients are exposed to a symptom-limited exercise test (on a bike in a controlled setting, with a safety protocol). Based on the achievements on this exercise test, patients started standard cardiac rehabilitation on a certain intensity level (low, moderate, or high intensity), or when randomized to OPTICARE XL CR started OPTICARE XL CR. The cycle ergometer, the rowing ergometer and fitness equipment were used for strength training. The training modalities for patients in the standard CR group consisted mainly of activities such as walking, jogging and group sports. During cardiac rehabilitation the patient is monitored every week and the training schedule is revised upon consultation with the multidisciplinary treatment team. Standard CR programs terminate once the patients’ physical and psychosocial recovery is sufficient. In most cases this goal is reached between 6 and 12 weeks, whereas the performance of the patient is evaluated in consultation with the multidisciplinary team. The OPTICARE XL CR program lasted for 12 weeks, since it was assumed that patients with obesity need at least 12 weeks of care in order to facilitate in behavioral changes. Furthermore, a behavioural after-care program was organized with 6 meetings (one hour each) between weeks 13–52 for the OPTICARE XL CR patients. This was done in small groups with a maximum of 8 participants instead of the standard CR groups with a maximum of 25 participants.

Weight loss was defined as any decrease in weight and measured with a calibrated weight scale. Clinically significant weight loss was defined as a loss of 5% of body weight at baseline [22]. Transthoracic echocardiography was performed at baseline and one-year follow-up. The study protocol was approved by the ’Medisch Ethische Toetsings Commissie Erasmus MC (MEC-2016-622)’ and written informed consent was obtained from all participants.

### Sample size calculation

Although there is some uncertainty regarding the standard deviations (SD) of the “delta’s” (changes from before to after the intervention) of the parameters that were studied (because these have not been reported in previous studies), these were expected to be smaller than the SD’s at either baseline or after the intervention reported in previous studies [23, 24], supporting the notion that this study will have sufficient power to detect changes in cardiac function caused by the intervention. The total sample size for the optional echocardiography substudy was aimed to be 50 patients, randomized to either the intervention or control group. When using a two-sided test in two groups of 25 patients, the study was powered to determine an effect size of 0.8 times the SD, regardless of the parameter studied (alpha 0.05 [two-sided], power 0.80).

### Transthoracic echocardiography

Two-dimensional grayscale harmonic images were obtained in the left lateral decubitus position using a commercially available ultrasound system (EPIQ 7, Philips, the Netherlands), equipped with a broadband (1-5 MHz) X5-1 transducer. Diastolic dysfunction and reduced ejection fraction (≤ 54% in females, and ≤ 52% in males) were defined according to the current guidelines [25, 26].

Interventricular septal thickness (IVSd), posterior wall thickness (PWd), and LV dimension (LVEDD) were all measured at end-diastole. The LV mass (LVM) was calculated according to the Deveraux formula using these measurements: LVM (g) = 0.80 × {1.04[(IVSd + LVEDD + PWd)^3^-(LVEDD)^3^]} + 0.6. LVM-index (LVMI) was calculated by dividing LVM by body surface area (BSA). BSA was calculated with the Mosteller formula [27]. LV hypertrophy (LVH) was defined as LVMI ≥ 102 g/m^2^ for males and ≥ 88 g/m^2^ for females [26].

**Fig. 1:**
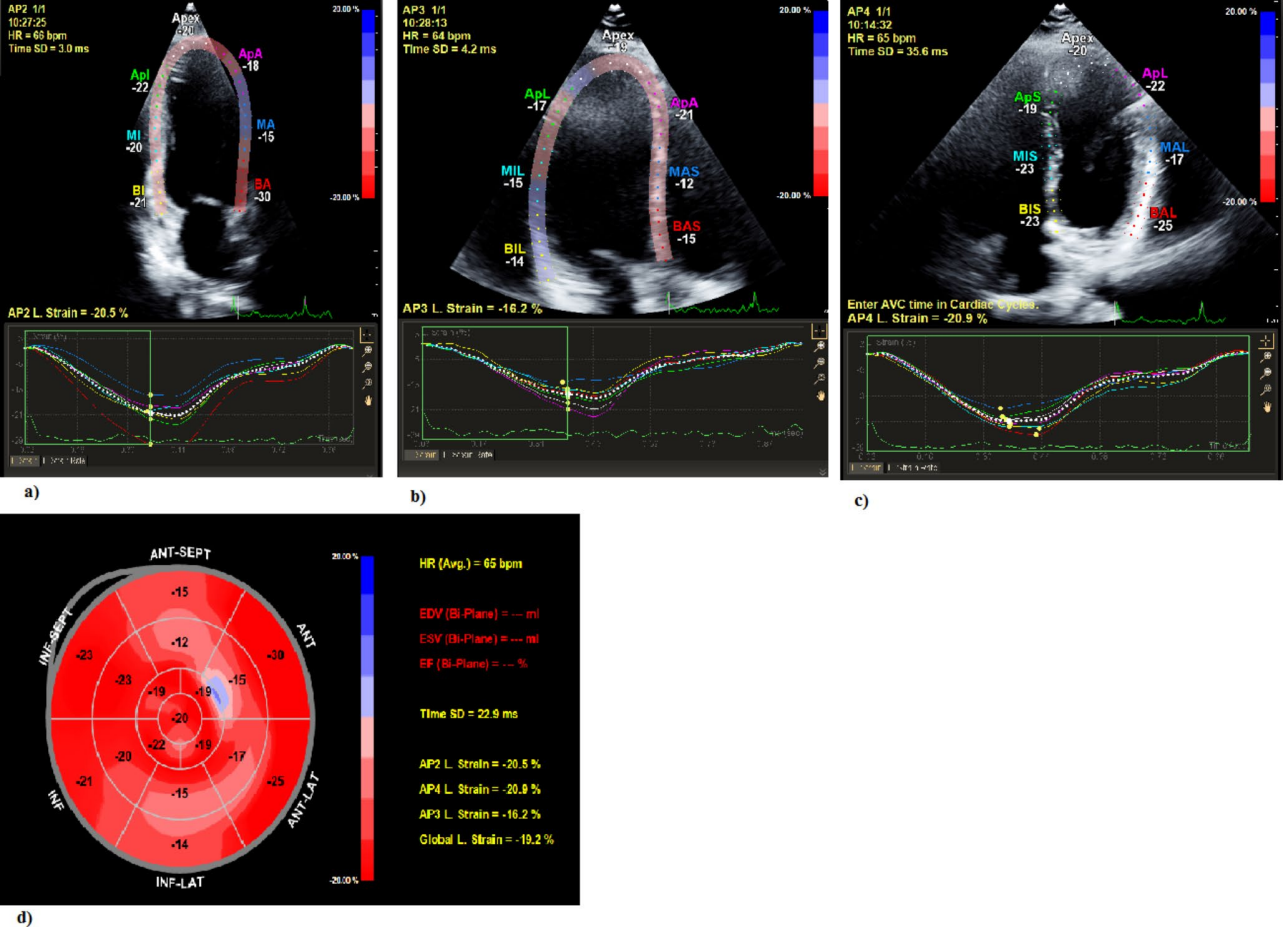
Measurement of global longitudinal strain (GLS) by speckle tracking analysis in an obesity patient (45 year old female, BMI 38.4 kg/m^2^). a) Apical 4-chamber view with measurement of longitudinal strain. b) Apical 2-chamber view with measurement of longitudinal strain. c) Apical 3-chamber view with measurement of longitudinal strain. d) Bull’s eye graph showing longitudinal strain for all myocardial segments, of which a weighted mean was used to derive GLS.

To optimize speckle tracking echocardiography, apical images were obtained at a frame rate of 60 to 80 frames/s. Three consecutive cardiac cycles were acquired from all apical views (4-chamber, 2-chamber, and 3-chamber). Subsequently, these cycles were transferred to a QLAB workstation (version 10.2, Philips, the Netherlands) for off-line speckle tracking analysis. Peak regional longitudinal strain was measured in 17 myocardial regions and a weighted mean was used to derive global longitudinal strain (GLS) (Fig. 1) [26]. GLS of lower than − 16% was considered abnormal [28].

### Statistical analysis

The normality of the data was checked by the Shapiro–Wilk test. The unpaired Student’s t-test for continuous variables was used to compare parameters between groups with normal distributions, the non-parametric Mann-Whitney U test for continuous parameters with skewed distributions, and the χ2 test/Fisher’s exact test for categorical variables. Continuous values were expressed as mean ± SD and categorical values as percentages.

Patients who completed follow-up were added to the following analyses. Repeated-measure analyses of variance (ANOVA) was used for continuous variables, to assess the relationship of changes at follow-up between groups. Generalized linear mixed models were used to compare the categorical data. The categorical parameters were entered subsequently as the dependent variable, and group (standard CR vs. OPTICARE XL CR) and follow-up as the independent variables. Random intercepts were used to account for the patient ID.

All statistical tests were 2-sided and a p-value of 0.05 was considered statistically significant. The analyses were performed with SPSS version 25 and R version 3.6.0 (glm package).

## Results

### Clinical characteristics and echocardiographic parameters of both groups at baseline

A total of 48 patients with obesity were included and randomized to both groups (standard CR n = 23, and OPTICARE XL CR n = 25). The inclusion of 50 patients was not achieved because the inclusion of the main study was completed at the time of the inclusion of 48 patients in the echocardiography substudy. The clinical characteristics of both groups are summarized in Table 1. The mean BMI was 34.8 kg/m^2^ in the standard CR group and 35.3 kg/m^2^ in the OPTICARE XL CR group (p = 0.69). Also, all other parameters of physical examination were comparable. Most comorbidities were comparable between groups, except that more patients in the standard CR group had hypercholesterolemia (p = 0.014). There were no differences in medication use between groups.

**Table 1:** Clinical characteristics and echocardiographic parameters of patients with obeisty in both groups at baseline

	Standard CR (n = 23 )	OPTICARE-XL CR (n = 25 )	p-value
**General characteristics**			
Age (years)	56 ± 8	58 ± 10	0.61
Male (n, %)	20 (87%)	19 (76%)	0.46
**Indication for rehabilitation**			
Coronary artery disease (n, %)	21 (91%)	19 (76%)	0.25
Atrial fibrillation (n, %)	2 (9%)	6 (24%)	0.25
**Physical examination**			
Length (m)	1.80 ± 0.11	1.77 ± 0.10	0.28
Weight (kg)	113 ± 15	111 ± 21	0.70
BMI (kg/m^2^)	34.8 ± 3	35.3 ± 5	0.69
BSA (m^2)^	2.38 ± 0.2	2.33 ± 0.3	0.50
Systolic BP (mmHg)	139 ± 19	135 ± 14	0.35
Diastolic BP (mmHg)	80 [75–90]	80 [75–89]	0.53
Waist circumference (cm)	119 ± 9	116 ± 12	0.30
Heart rate (bpm)	66 ± 10	67 ± 10	0.58
**Comorbidity**			
Diabetes Mellitus (n, %)	5 (22%)	5 (20%)	0.88
Hypertension (n, %)	11 (48%)	9 (36%)	0.41
Hypercholesterolemia (n, %)	10 (44%)	3 (12%)	**0.014**
History of smoking (n, %)	19 (86%)	18 (72%)	0.23
COPD (n, %)	1 (4%)	2 (8%)	0.60
OSAS (n, %)	1 (4%)	3 (12%)	0.34
**Medication**			
Beta-blockers (n, %)	12 (57%)	19 (79%)	0.11
ACE inhibitors (n, %)	14 (61%)	21 (84%)	0.09
Statins (n, %)	17 (74%)	18 (72%)	0.63
Antiarrhythmic drugs (n, %)	4 (17%)	1 (4%)	0.11
Insulin (n, %)	3 (13%)	1 (4%)	0.23
Oral anti-diabetics (n, %)	10 (43%)	7 (28%)	0.20
**Echocardiography parameters**			
LVM (g)	207 [173–255]	242 [183–334]	0.11
LVM-index (g/m^2^)	90 [70–111]	99 [86–125]	**0.029**
Left ventricular hypertrophy (n,%)	7 (30%)	14 (56%)	0.07
Mitral inflow E-wave (cm/s)	68 ± 12	62 ± 17	0.14
Mitral inflow A-wave (cm/s)	69 ± 15	67 ± 16	0.68
E/A-ratio	0.94 [0.86–1.20]	0.90 [0.75–1.10]	0.29
Septal e’ velocity (cm/s)	8 ± 2	7 ± 2	0.05
Lateral e’ velocity (cm/s)	10 ± 2	9 ± 2	0.19
E/e’-ratio	9 ± 2	9 ± 3	0.62
Deceleration time (s)	0.19 [0.18–0.23]	0.19 [0.15–0.22]	0.43
LA volume index (ml/m^2^)	28 [24–35]	26 [23–35]	0.67
TR velocity (cm/s)	93 [77–131]	94 [81–179]	0.69
Diastolic dysfunction (n, %)	10 (43%)	16 (64%)	0.15
TAPSE (cm)	2.4 ± 0.4	2.4 ± 0.5	0.97
LV ejection fraction (%)	55 ± 8	53 ± 9	0.57
Reduced LV ejection fraction (n,%)	8 (35%)	12 (48%)	0.35
GLS (%)	-17 ± 3	-16 ± 3	0.27
Reduced GLS (n,%)	8 (35%)	11 (46%)	0.44

Values represent mean ± standard deviation, median (Q1-Q3) or n (%)

*BMI* = body mass index, *BSA* = body surface area, *COPD* = chronic obstructive pulmonary disease, *CR* = cardiac rehabilitation, *OSAS* = obstructive sleep apnea syndrome, *ACE* = angiotensin converting enzyme, *LVM* = left ventricular mass, *E-wave* = early diastolic transmitralflow velocity, *A-wave* = late diastolic transmitralflow velocity, *e’*=early diastolic mitral annular velocity, *LA-volume index* = left atrial volume index, *TR* = tricuspid regurgitation, *TAPSE* = tricuspid annular plane systolic excursion, *GLS* = global longitudinal strain

Almost all echocardiographic parameters were comparable between groups, except for the LVM-index, which was increased in the OPTICARE XL CR group (99[86–125]g/m^2^ vs. 90[70 − 11]g/m^2^, p = 0.029). However, the percentage of patients with LVH was not significantly different between groups (56% vs. 30%, p = 0.07).

### One-year follow-up; all patients

**Fig. 2:**
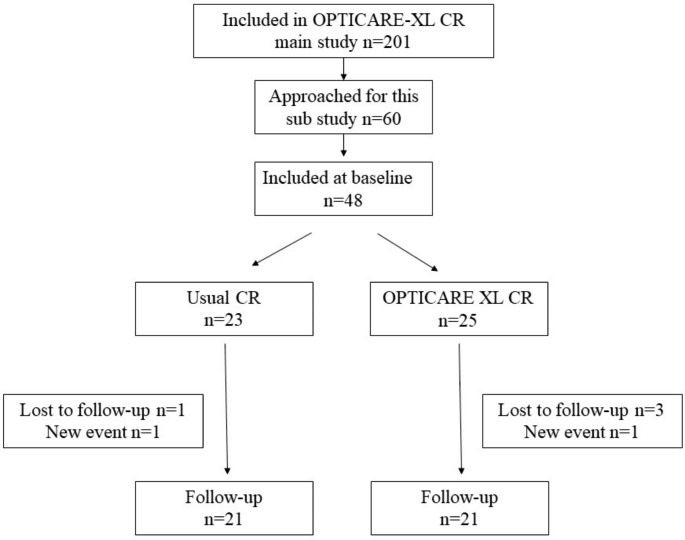
Flow chart. (Standard CR = standard cardiac rehabilitation)

Four patients did not complete the follow-up and two patients had a new cardiac event. A total of 42 patients completed the one-year follow-up (Fig. 2). Table 2 shows that there was significant weight loss at follow-up within both study groups (p < 0.001). Also, the heart rate increased (p = 0.032). None of the echocardiographic parameters changed from before to one year after either form of rehabilitation.

**Table 2:** Physical examination and echocardiographic parameters at baseline and at one-year follow-up

	Standard CR (n = 21 )		OPTICARE-XL CR (n = 21 )			
	Baseline	Follow-up	Baseline	Follow-up	p-value baseline vs. follow-up	p-value* usual CR vs. OPTICARE-XL CR
**Physical examination**						
Weight (kg)	113 ± 15	110 ± 17	111 ± 21	105 ± 22	**0.001**	0.43
BMI (kg/m^2^)	34.8 ± 3	33.9 ± 4	35.3 ± 5	33.5 ± 5	**< 0.001**	0.45
Heart rate (bpm)	65 ± 9	67 ± 11	68 ± 13	72 ± 17	**0.032**	0.41
**Echocardiographic parameters**						
LVM (g)	219 ± 60	207 ± 67	257 ± 80	237 ± 75	0.17	0.49
LVM-index (g/m^2^)	92 ± 22	88 ± 27	110 ± 30	117 ± 66	0.80	0.25
Left ventricular hypertrophy (n,%)	7 (33%)	5 (24%)	14 (67%)	10 (48%)	0.63	**0.019**
Mitral inflow E-wave (cm/s)	68 ± 12	65 ± 14	69 ± 14	64 ± 17	0.82	0.77
Mitral inflow A-wave (cm/s)	69 ± 16	63 ± 13	66 ± 14	65 ± 14	0.78	0.27
E/A-ratio	1.03 ± 0.32	1.01 ± 0.19	1.01 ± 0.34	0.95 ± 0.26	0.27	0.66
Septal e’ velocity (cm/s)	8 ± 2	7 ± 2	8 ± 2	8 ± 3	0.27	0.12
Lateral e’ velocity (cm/s)	10 ± 2	10 ± 2	11 ± 2	9 ± 3	0.67	0.57
E/e’-ratio	8.8 ± 2	9.7 ± 2	8.8 ± 2	8.4 ± 2	0.11	0.13
Deceleration time (s)	0.20 ± 0.04	0.19 ± 0.06	0.20 ± 0.04	0.19 ± 0.06	0.70	0.79
LA volume index (ml/m^2^)	30 ± 8	30 ± 9	30 ± 8	32 ± 8	0.44	0.36
TR velocity (cm/s)	110 ± 58	138 ± 72	113 ± 61	155 ± 81	0.37	0.52
TAPSE (cm)	2.4 ± 0.4	2.4 ± 0.6	2.3 ± 0.4	2.4 ± 0.6	0.40	0.19
Diastolic dysfunction (n, %)	9 (43%)	8 (38%)	14 (67%)	13 (62%)	0.56	0.07
LV ejection fraction (%)	55 ± 9	54 ± 8	54 ± 8	53 ± 10	0.49	0.88
Reduced LV ejection fraction (n,%)	7 (33%)	7 (33%)	10 (48%)	9 (43%)	0.75	0.28
GLS (%)	-17 ± 3	-16 ± 3	-16 ± 3	-16 ± 5	0.38	0.66
Reduced GLS (n,%)	7 (33%)	9 (43%)	9 (43%)	11 (52%)	0.44	0.38

Values represent mean ± standard deviation or n (%)

* p-value of the changes over time, comparison between groups

p-values displayed of continuous variables were calculated with repeated measures ANOVA, and for the categorical variables with generalized linear mixed models

*BMI* = body mass index, *CR* = cardiac rehabilitation, *GLS* = global longitudinal strain, *LV* = left ventricular, *LVM* = left ventricular mass, *LVM-index* = left ventricular mass index, *TAPSE* = tricuspid annular plane systolic excursion, *TR velocity* = tricuspid regurgitation velocity

### One-year follow-up; comparison between groups

**Fig. 3:**
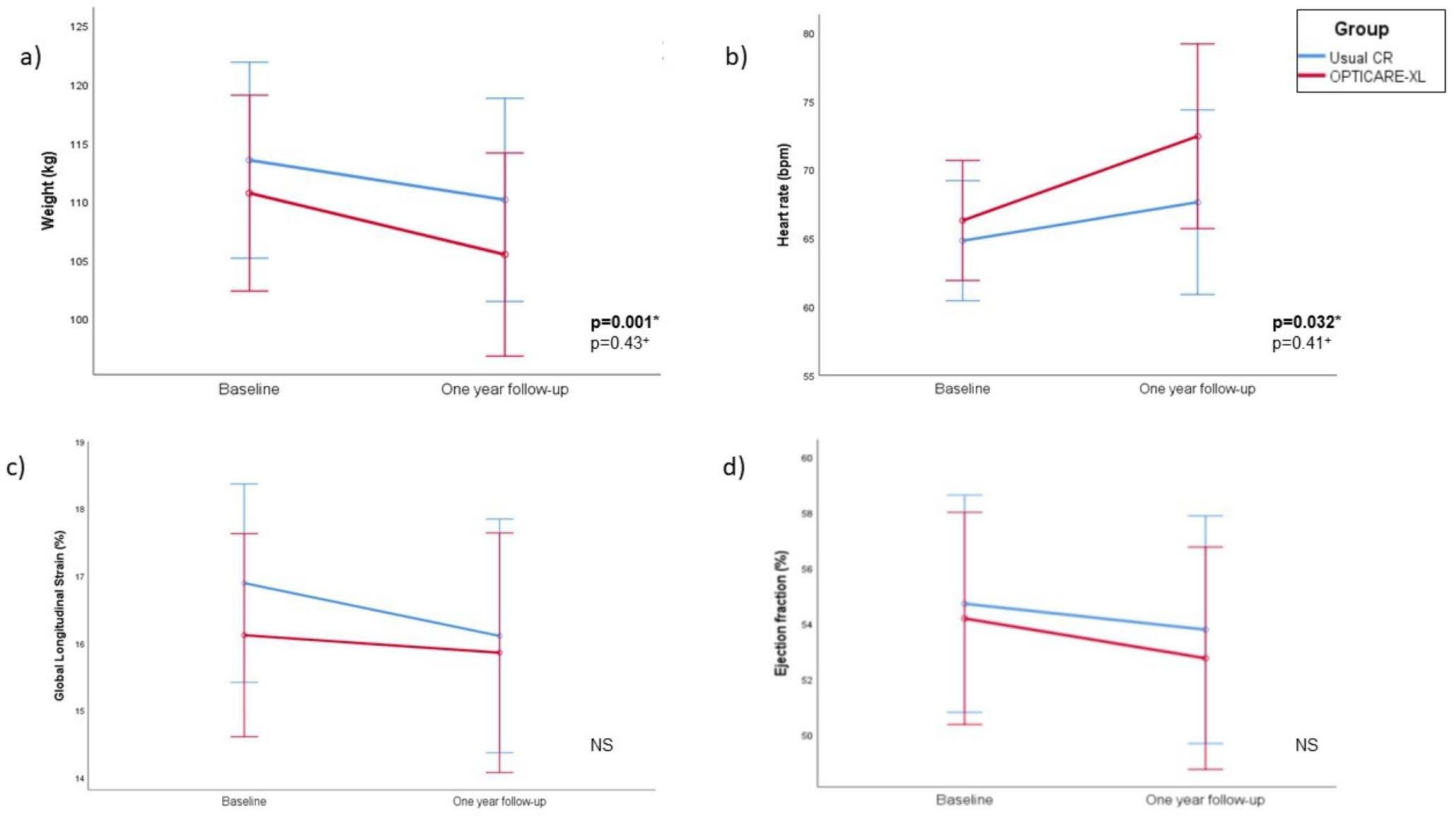
Changes pre- and post-cardiac rehabilitation in mean weight (a), heart rate (b), global longitudinal strain (c), and ejection fraction (d). (Error bars represent 95% CI, Significant at p < 0.05, NS = non significant)

**Fig. 4:**
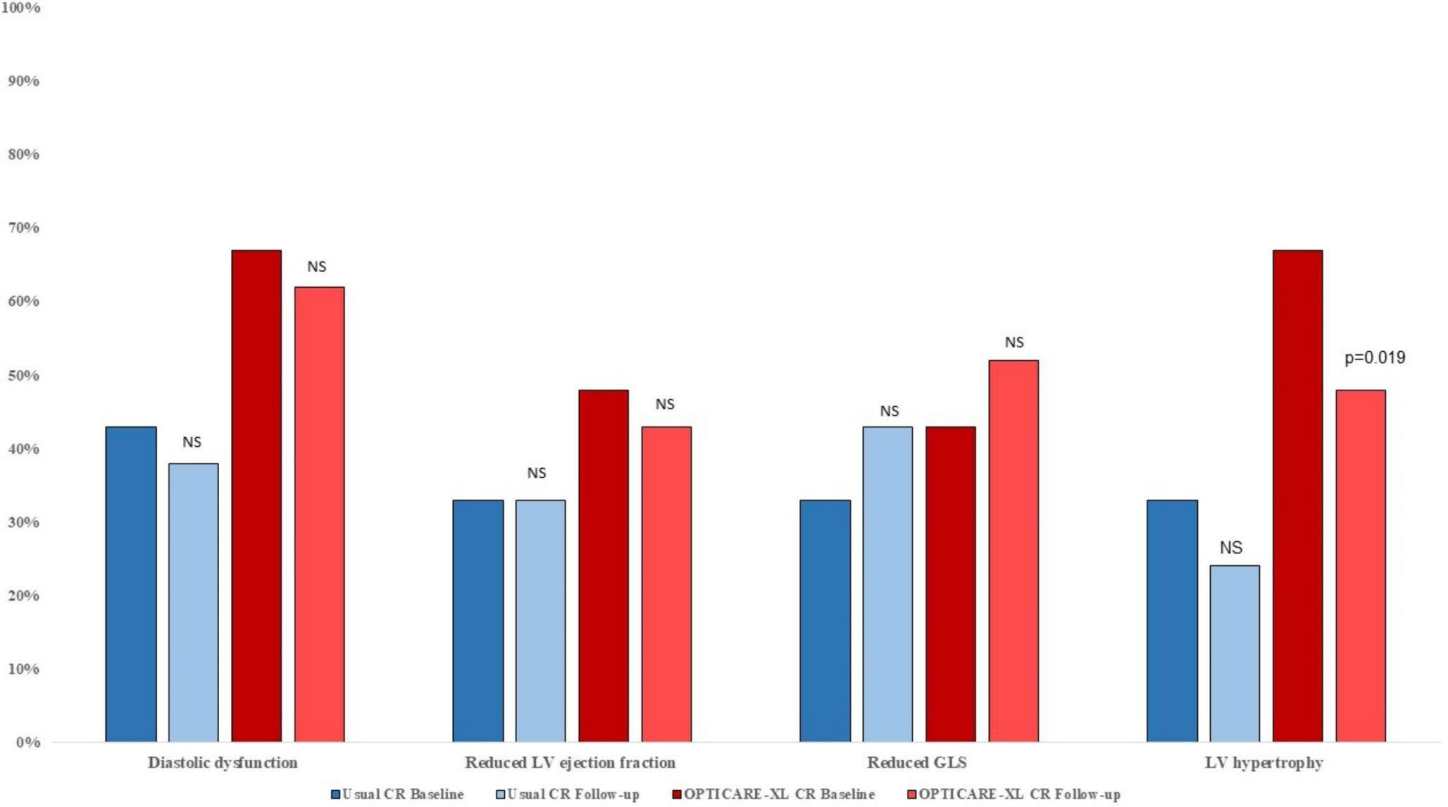
Comparison of percentage of diastolic dysfunction, reduced ejection fraction, reduces global longitudinal strain, and left ventricular hypertrophy between both standard cardiac rehabilitation, OPTICARE XL CR, and baseline and follow-up. (LV = left ventricular)

Weight loss was comparable between groups (Table 2; Fig. 3). A total of 13 patients in the standard CR group lost weight, as compared to 16 of the patients in the OPTICARE XL CR group (p = 0.43). Clinical significant weight loss was achieved in 5 patients in the standard CR, and in 7 in the OPTICARE XL CR group (p = 0.50). Also, the increase in heart rate that was present in the total group was comparable between groups (p = 0.41). The vast majority of changes in echocardiographic parameters were comparable as well. Only the proportion of patients with LVH was more decreased in the OPTICARE XL CR group at one-year follow-up (p = 0.019) (Fig. 4).

## Discussion

The main finding of the current study is that cardiac function as measured with echocardiography in patients with obesity did not improve one-year after a novel state of the art CR program (OPTICARE XL CR) as compared to standard CR.

Although there was a significant and promising reduction in weight and thereby BMI in both groups at one-year follow-up, this reduction was relatively small and does not meet the definition of clinically significant weight loss [22] This finding of mild weight reduction one year after CR is consistent with the results of other studies that focused on a shorter term [29, 30] In a study of 73 patients with obesity and coronary artery disease also a statistically significant but small decrease in weight was observed after completing CR [30] Also, a larger registry containing data of 29,601 patients with obesity in the UK found a small (mean 0.9 kg) but significant weight loss after CR [31] In another study it was concluded that the prevalence of obesity did not change significantly after the CR program (37–33%), and the prevalence of severe obesity (BMI > 35 kg/m^2^) improved only slightly (3.5–2.5%).[29]^29^ Our outcomes suggest that a significant clinical weight reduction through CR seems difficult to achieve for the majority of patients, even with interventions that are specifically designed for this population.

The current study is the first in which echocardiographic parameters of cardiac function were investigated in patients with obesity who participated in standard CR or in a CR program specifically designed for patients with obesity. However, a few small studies have been performed in patients with obesity in which the impact of *exercise* on cardiac function as measured by speckle tracking echocardiography was evaluated. Schuster et al. [23] described a GLS of -15.9 ± 0.8% in 10 patients with obesity at baseline, significantly improving to -17.4 ± 0.9% after 8 weeks of training. The training program consisted of three 45-min sessions of walking and/or cycling at home, without any dietary intervention. The body fat mass decreased without significant weight or BMI change by this intervention. In another study, Obert et al. [24] evaluated the impact of both diet and exercise training in 28 adolescents with obesity. The training consisted of nine sessions of 5-min each, three times per week. Additionally, moderate physical activities were performed twice a week during the first two months and then five times per week during the following 7 months. Moreover, the total daily calorie intake was controlled at about 2300-2500 kcal. Both body weight (99 ± 15 kg vs. 84 ± 115 kg) and BMI (36 ± 5 kg/m^2^ vs. 31 ± 5 kg/m^2^) decreased significantly. The GLS in these patients was − 14.2 ± 3.6% at baseline, and improved significantly to -16.9 ± 3.5% at 9 months follow-up. In contrast, in our study, GLS did not improve in patients with obesity after one year of either program. An explanation for this could be the more intense exercise training in the mentioned other studies. Also, follow-up in our study was one year, which is considerably longer than the follow-up in the aforementioned studies. Therefore, it may be hypothesized that weight loss and concurring improved GLS at the beginning of CR was higher in our patients as well, but that the effect was lost later due to failure to maintain weight loss.

There are several studies in which echocardiographic parameters of LV function, such as GLS, were assessed in patients who participated in standard CR. However, the focus of none of the studies was on patients with obesity. First, Malfatto et al. [32] studied GLS is a small group who entered CR after a first uncomplicated myocardial infarction. They concluded that a short period of intensive CR induces rapid recovery of GLS (-17.0 ± 3.7% vs. -20.1 ± 3.2%) and diastolic function. Also, Acar et al. [33] found a significant increase in LV ejection fraction (49 ± 7.9% vs. 54 ± 9.1%) and GLS (-13 ± 2.3% vs. -17 ± 3.0%) after three months of standard CR in a group of 27 patients with acute myocardial infarction. We found no changes in GLS and LV ejection fraction. Our study was different as compared to these other studies regarding the focus specifically on patients with obesity and the longer follow-up. As mentioned earlier, patients with obesity have a higher cardiometabolic risk and poorer fitness [12], which could also have a negative impact on GLS recovery. In a recent study, it was shown that decreased baseline fitness, such as may be present in patients with obesity, may moderate weight loss achieved by weight loss programs [34]. However, despite this, we did expect that echocardiographic parameters of cardiac function such as GLS would improve after OPTICARE XL CR, which unfortunately did not happen.

Finally, both groups showed a mild but non-significant decrease of LVH after rehabilitation. Nevertheless, the proportion of patients with LVH was significantly more decreased in the OPTICARE XL CR group. This finding suggests that the more intensive program of the OPTICARE XL CR may have a positive contribution to the reduction of LVH. However, more research is needed to conform this.

### Limitations

Although echocardiographic parameters of cardiac function did not improve in patients undergoing CR in our study, one may hypothesize that such parameters would even have worsened without any CR. However, because a control group of patients with obesity not undergoing any CR is lacking in the current study, this remains to be investigated. The number of subjects in this study was small. Studies with a larger sample size would be necessary for further evaluation of some of the trends that have been observed. Also, a more intensified echocardiographic follow-up with measurements for example also at six months would have allowed better comparison with the other studies with shorter follow-up described before. Nevertheless, the long term follow-up is also a major strength of our study.

## Conclusion

Both in patients with obesity undergoing standard CR or a novel CR program designed for patients with obesity, echocardiographic parameters of cardiac function did not improve at one-year follow-up. To our knowledge, this is the first publication describing findings of extensive echocardiographic evaluation before and after tailor-made cardiac rehabilitation for patients with obesity.
